# Differences in Accumulation and Virulence Determine the Outcome of Competition during *Tobacco etch virus* Coinfection

**DOI:** 10.1371/journal.pone.0017917

**Published:** 2011-03-15

**Authors:** Guillaume Lafforgue, Josep Sardanyés, Santiago F. Elena

**Affiliations:** 1 Instituto de Biología Molecular y Celular de Plantas, Consejo Superior de Investigaciones Científicas – UPV, València, Spain; 2 Santa Fe Institute, Santa Fe, New Mexico, United States of America; Institute of Infectious Disease and Molecular Medicine, South Africa

## Abstract

Understanding the evolution of virulence for RNA viruses is essential for developing appropriate control strategies. Although it has been usually assumed that virulence is a consequence of within-host replication of the parasite, viral strains may be highly virulent without experiencing large accumulation as a consequence of immunopathological host responses. Using two strains of *Tobacco etch potyvirus* (TEV) that show a negative relationship between virulence and accumulation rate, we first explored the evolution of virulence and fitness traits during simple and mixed infections. Short-term evolution experiments initiated with each strain independently confirmed the genetic and evolutionary stability of virulence and viral load, although infectivity significantly increased for both strains. Second, competition experiments between hypo- and hypervirulent TEV strains have shown that the outcome of competition is driven by differences in replication rate. A simple mathematical model has been developed to analyze the dynamics of these two strains during coinfection. The model qualitatively reproduced the experimental results using biologically meaningful parameters. Further analyses of the model also revealed a wide parametric region in which a low-fitness but hypovirulent virus can still outcompete a high-fitness but hypervirulent one. These results provide additional support to the observation that virulence and within-host replication may not necessarily be strongly tied in plant RNA viruses.

## Introduction

RNA viruses are among the most common pathogens of plants, and their evolution has been studied experimentally and phylogenetically, as well as with theoretical and computational models. Plant viruses have been used as model systems for exploring the mechanisms of virus evolution [1], [2]. A few peculiarities of plant viruses, compared with their animal and bacterial counterparts, and that arise as consequence of host's properties, are: (*i*) plants cells have walls whose connections are restricted and a successful virus must evolve to move throughout plasmodesmata and reach phloem to systemically colonize the plant, (*ii*) this also implies that not all viral particles produced are released and have the opportunity to infect new cells, thus relaxing selection for beneficial mutations, (*iii*) plants are sessile organisms so virus must also be able to transmit from host to host with the intervention of a third player, the transmission vector, and (*iv*) plants do not have immune system but instead have both specific and non-specific defense responses to viruses [3]. Like their animal relatives, plant RNA viruses have the potential to establish very high population diversity, because of their error-prone replication and short generation times. Consequently this property leads them to rapid evolution and great evolvability [4].

Virulence, which can be defined as the deleterious effects of parasites on their hosts, is a selectable trait and thus, could play an important role in the evolution of pathogens [5]. Because virulence does not represent any clear advantage for parasites, which depend on their hosts for survival and spreading, it is not obvious why parasites harm their hosts. A commonly accepted hypothesis is that virulence is an unavoidable consequence of parasite multiplication within the infected hosts [6], [7]. Under this assumption, the evolution of pathogens would be subjected to a tradeoff between virulence and transmission [8]. Therefore selection within and between hosts would result in a level of virulence that optimizes both multiplication and transmission of the pathogen [9], [10].

Experimental support for a positive correlation between within-host multiplication rates and virulence is limited for plant-virus systems. However, it has been shown that a positive correlation between parasite multiplication and virulence may exist only in some genotypes and/or environmental conditions for a given host-parasite system [7]. Therefore, the virulence of RNA viruses depends both on the host genotype and the virus genome. To illustrate this, a study with *Cucumber mosaic virus* (CMV) demonstrated a relationship between virulence and host genotype independent of virus multiplication [11]. No correlation between virus accumulation and symptoms severity was detected. Moreover changes observed in virulence during horizontal and vertical transmission experiments with *Barley stripe mosaic virus* were not due to changes in virus accumulation [12]. Evidence for a positive relationship between parasite multiplication and virulence comes mostly from microparasites infecting animals [7], [13].

Coinfection assays allow for the study of competition dynamics between different viral genotypes, and can be useful to determining what are the main forces involved in their long-term fate. For instance, using two strains that differ in virulence and multiplication rate. The outcome of such competition determines the genetic structure of the viral population and, therefore, the level of competition, as well as the phenotypic properties of such population (e.g., virulence). Theoretical models of multiple infections in which selection of parasites occurs thought competition for multiplication within host and for transmission among host have been studied [14]. Two extreme situations are usually considered: (*i*) the most virulent parasite will outcompete the others within the host and (*ii*) coinfecting viruses do not compete. Mosquera and Adler [15] have proposed a coinfection model that considers competition between two parasites, which may affect their transmission rate, and the possibility that the most virulent takes over the host. The short–sighted explanation for the evolution of virulence postulates that, during multiple infections, competition for resources selects strains that have the best rate of multiplication; higher virulence is a side effect of fast replication [16].

*Tobacco etch virus* (TEV), the pathogen employed in this study, induces symptoms that range from chlorotic vein banding, mosaic mottling, necrosis and/or distortion of leaves in susceptible dicotyledonous species. Flowers, seeds and fruits are also affected. TEV has a positive sense, single strand RNA genome and taxonomically has been classified in the genus *Potyvirus* within the *Potyviridae* family. The effects on TEV fitness and virulence of random single-nucleotide substitutions have been recently characterized [17]. Most mutants have a reduced fitness relative to the wildtype virus. However, mutational effects on virulence are more variable, ranging from hyper- to hypovirulent [17]. No significant correlation exists between these two traits. Therefore, adaptive evolution of TEV (i.e., associated with within-host fitness increases) may result in widely different virulence levels.

The existence of a hypervirulent strain TEV-PC2 with a low accumulation rate and of a hypovirulent strain TEV-PC76 with a high accumulation rate [17] opens the possibility of exploring how virulence evolves as a consequence of the competition between pathogens for which no positive association exists between virulence and within-host accumulation. What trait determines the result of coinfection the most, differences in virus replication or differences in symptoms severity? To shed light on this question, we have evaluated the within-host multiplication of the TEV-PC2 and TEV-PC76 strains in single and double infections in the natural host *Nicotiana tabacum*. To better interpret the experimental results, we developed a mathematical model that describes the temporal dynamics of two coinfecting viruses differing in their accumulation rate and virulence in the same way that our experimental subjects do. We first used the model to explain qualitatively the experimental results using biologically meaningful parameters, also characterizing the equilibrium values for the scenarios of coexistence, outcompetition and coextinction. The model also showed that under certain combinations of parameters a slow replicating hypovirulent strain can outcompete a fast replicating hypervirulent one.

## Results

### Short-term stability of virulence and fitness traits during single infections

**Figure 1:**
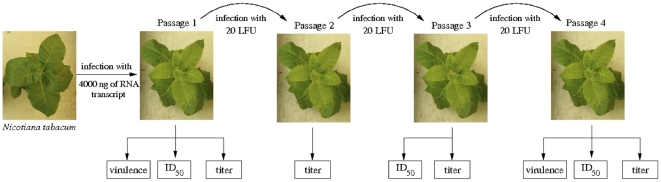
Scheme of the experimental evolution procedure and virulence and *ID*_50_ (infectivity) estimation. Seven dpi, virus accumulation (titer) was evaluated by local-lesion assays on *C. quinoa* and then concentrations were made equal so each newly infected plant received 20 LFU per evolutionary passages or to 30 LFU/µL for *ID*_50_ determination.

**Figure 2:**
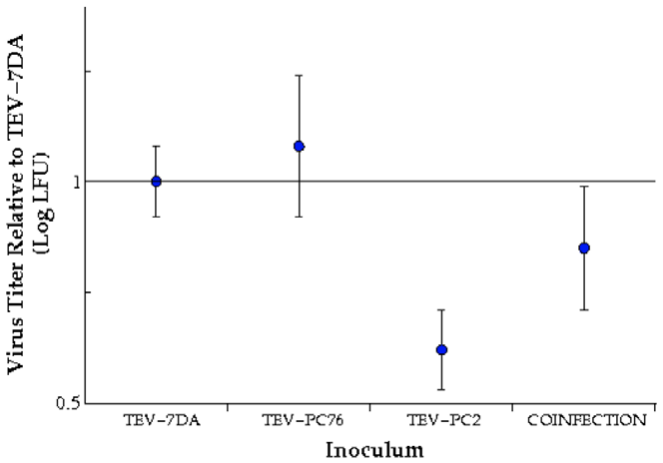
Mean virus accumulation values (± SD) relative to the average value estimated for the wildtype TEV-7DA. Values correspond to averages across replicate lineages for single infections and coinfections.

First, we sought to determine whether the phenotypic properties of strains TEV-PC2 and TEV-PC76 were stable after short periods of evolution. To do so, eight independent lineages were initiated for each virus and serially transferred every 7 dpi ensuring that the same amount of LFU was used to initiate each new infection. Virus accumulation, infectivity and virulence were evaluated at each passage (Fig. 1). Virulence was an evolutionarily stable trait that did not change after the four passages (*F*_1,36_ = 0.178, *P* = 0.676), and the differences in virulence between TEV-PC2 and TEV-PC76 were maintained along the experiment (*F*_2,36_ = 205.379, *P*<0.001), with the former strains being, on average, 9.84% more virulent than the later. Evolutionary stability was also observed for viral accumulation. While the TEV-PC2 strain viral accumulation was, overall, significantly lower than for TEV-PC76 (*F*_2,66_ = 16.352, *P*<0.001), the differences in accumulation among the two strains remained significant along the evolution experiment (*F*_2,66_ = 16.352, *P*<0.001): the TEV-PC76 accumulates 74% more than TEV-PC2 per gram of infected tissue. Interestingly, TEV-PC76 accumulation was undistinguishable from that of the wiltype strains (*post hoc* Tukey test, *P* = 0.682). Virus accumulation values can be normalized by the average value observed for the wildtype TEV as 1.07 for TEV-PC76 and, similarly, down to 0.621 for TEV-PC2 (Fig. 2).

Contrarily to these observations, overall infectivity significantly increased with passages for both strains (*F*_1,38_ = 15.864, *P*<0.001), although the magnitude of the difference between them remained constant (test of interaction: *F*_1,38_ = 0.041, *P* = 0.840), being TEV-PC76 7.04% more infectious than TEV-PC2 along the evolution experiment.

### Competition between TEV-PC2 and –PC76 viral strains

In the case of mixed infections, virus accumulation was intermediate between values characteristic of each strains but significantly grouped to the TEV-PC76 values (Fig. 2; *post hoc* Tukey test, *P* = 0.133), suggesting a dominance of this strain in determining overall virus accumulation. These results are consistent with those previously reported by Carrasco *et al.* [17] quantifying relative fitness by means of competition experiments.

**Figure 3:**
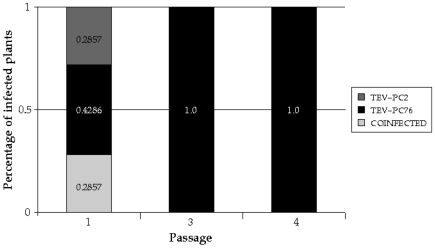
Percentage of infected plants infected by one or the two viral strains as determined by the RT-PCR/restriction analysis.

Next, we determined the composition of viral populations on each coinfected plant 7 dpi by means of the RT-PCR followed by diagnostic restriction analyses. Only ∼43% of the coinoculated plants were diagnosed as coinfected (Fig. 3). In some cases, only TEV-PC2 was detected by this method. In cases of no coinfection, one may conclude that (*i*) one virus was completely outcompeted by its counterpart and is not present in the plant anymore or (*ii*) it may still be present but at a concentration that is under the method detection level. To determine what of these two options was correct, we made virus preparations from all plants, regardless their infectious status, and used them to continue the evolution experiment. At passage three, however, all population switched radically into a strict TEV-PC76 hypovirulent population, suggesting that the second possibility was, indeed, the case. Furthermore, this dominance of the TEV-PC76 strain is consistent with previous results from head-to-head competition assays against the wildtype virus showing that the hypervirulent TEV-PC2 had lower competitive fitness than the hypovirulent TEV-PC76 [17]. Knowing this, and assuming that fitness values are transitive [18], we conclude that TEV-PC76 is a better competitor than TEV-PC2 as a consequence of its larger accumulation and independently of its hypovirulent phenotype.

### A mathematical model for the competition between hypo- and hypervirulent strains

The previous results indicate that a hypervirulent virus that reaches low viral accumulation is outcompeted by a hypovirulent one but that it reaches high accumulation. In order to disentangle the effects between accumulation and virulence we developed a dynamical mathematical model for virus competition together with differential virulences. The model was formulated by means of a two-species time-continuous dynamical system considering as state variables two populations of viruses, named *x*_1_ and *x*_2_. The model is similar to the one analyzed by Solé *et al.* [19] but with the difference that we also included the effect of virulence on the dynamics. The model assumes infinite diffusion and no stochasticity and is given by the next two coupled autonomous differential equations:
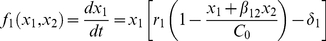
(1)

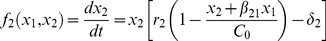
(2)with
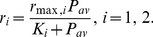
(3)


In order to introduce virulence we assumed that viruses need a cellular factor, *P*, to complete its reproductive cycle (e.g., ribosomes) and that the utilization of such factor by the virus translates into a defects in cell cycle and thus in symptoms. We defined the available quantity of such cellular factor as 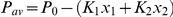. Note that *P_av_* decreases as viral populations grow in size. We assumed *P*_0_ = 1 to be a mean and constant concentration value of the limiting cellular factor. *K_i_* (*i* = 1, 2) are the affinity constants for the limiting cellular factor, and *r_i_* the realized growth rates for the *i* ^th^ viral strain, being *r_max,i_* the maximum replication rate when the cellular factor is present at infinite concentration and is not limiting viral growth. We would like to highlight that the model assumes that the virus accumulation is entirely determined by replication rate. This assumption may not be entirely realistic from a virological perspective, since accumulation may also depend of other factors such as cell-to-cell and systemic movements, but it is convenient from the mathematical point of view, since it avoids considering spatial correlations, and, in addition, does not require of knowledge about viral spread, which has not been gathered in the above experiments. In our formulation the virulence of a given strain *i* is proportional to *K_i_* and this proportionality creates the observed tradeoff between accumulation rate and virulence shown in Eq. (3). We assumed that both viral strains compete in a finite bounded system (e.g., in the plant or in a plant tissue), using a logistic-like constraint (with carrying capacity *C*_0_, hereafter is scaled to *C*_0_ = 1) that couples both populations and introduces a competition term associated to the growth inside the host. The parameter *β_ij_* in the logistic term corresponds to the interspecific competition rates. As previously noted, the experimental results suggested no major interference between both viruses, therefore we considered symmetric interspecific competition i.e., *β_ij_* = *β_ji_*≡*β*, and for simplicity we hereafter used *β* = 1. Finally, we assumed degradation rates (*δ_i_*>0; *i* = 1, 2) to be symmetric i.e., *δ*_1_ = *δ*_2_≡*δ*. Following the previous empirical observations, the model does not consider changes in virulence and virus replicative fitness along time.

We analytically and numerically studied Eqs. (1) and (2). All numerical results were performed solving the differential equations with the fourth-order Runge-Kutta method (with a constant stepsize Δ*t* = 0.1). The terms inside the Jacobian matrix for this dynamical system,
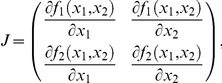
are given by
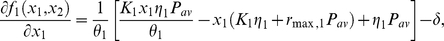


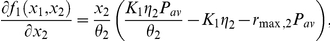


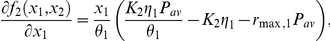
and
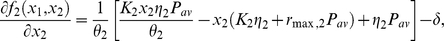
with *θ_i_* = *K_i_*+*P_av_*, and *η_i_* = *r_max,i_*(1−*x*_1_−*x*_2_), with *i* = 1, 2.

**Figure 4:**
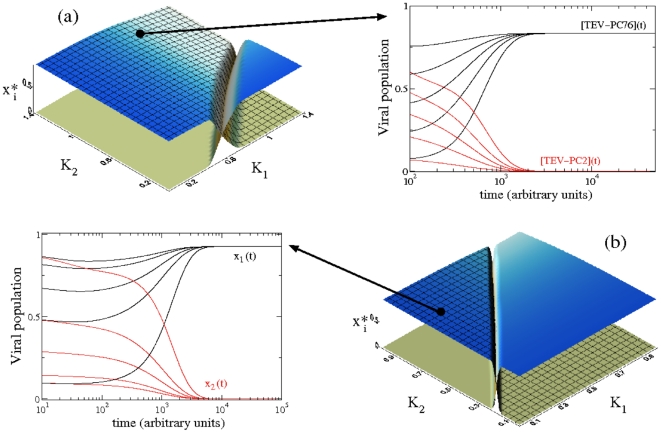
Dependence of the outcompetition dynamics on the fitness of each virus population (*r_max,i_*) and on the affinities to the cellular factor *K_i_* (i.e., virulence). (a) Equilibrium concentration numerically obtained for *x*_1_ (gridded surface) and *x*_2_ (flat surface) shown in the parameter space (*K*_1_, *K*_2_) using the mean values of fitness experimentally characterized: *r_max_*_,1_ = 1.07 (TEV-PC76) and *r_max_*_,2_ = 0.621 (TEV-PC2), using *x*_1_(0) = *x*_2_(0) = 0.5. The dynamics is shown on the right hand side using the virulence parameters characterized for the same strains in Carrasco *et al.* (2007), which are indicated by the large arrow and given by: *K*_1_ = 0.818 (TEV-PC76, black trajectories for *x*_1_) and *K*_2_ = 1.221 (TEV-PC2, red trajectories for *x*_2_). Note that *x*_1_ asymptotically outcompetes *x*_2_, independently of the initial condition. (b) Same as in (a) but using *r_max_*_,1_ = 0.85 and *r_max_*_,2_ = 1. Note that for this case, as in the previous one, the hypovirulent virus can displace the hypervirulent one, even if the former has a lower replicative fitness. The time series show, for five different initial conditions, the dynamics for the values of virulence indicated with the arrow, given by *K*_1_ = 0.2 and *K*_2_ = 0.7.

Equations (1)–(2) have six fixed points. As we were interested in the scenarios of extinction and of outcompetition, we focused our analyses in three fixed points: one involving the extinction of both strains and the two equilibrium points involving the extinction of one viral strain and the survival of the other one. Together with these three equilibria, there is another fixed point that can involve the coexistence of both viral strains (see below), as well as two other fixed points that involve the outcompetition of one of the two strains. However, numerical investigations for these latter two equilibria indicate that, under the parameter regions we are studying (Fig. 4), the non-trivial values for such points are outside the biologically meaningful parameters (i.e., *x_i_* ^*^>1, results not shown) imposed by the logistic-like constraint (with carrying capacity *C*_0_ = 1), and thus are not analyzed. The first fixed point is the trivial equilibrium, *P*_1_^*^ = (*x*_1_^*^ = 0, *x*_2_^*^ = 0), where both strains have zero population numbers. The stability of such a point is obtained by linearizing the flow and computing the eigenvalues from det(*J*(0)−*λ* **I**) = 0, with
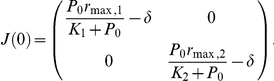
The eigenvalues, obtained directly from the diagonal, are given by:
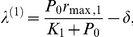
and
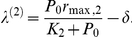
Note that the trivial fixed point is stable when 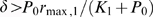 and 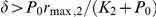. The other two biologically meaningful equilibrium points, which are responsible of the outcompetition of one of the virus population, are denoted by *P*_2_^*^ = (*x*_1_^*^ = *Γ*, *x*_2_^*^ = 0) and *P*_3_^*^ = (*x*_1_^*^ = 0, *x*_2_^*^ = *Λ*), with
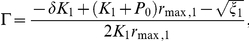


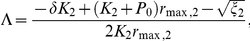
with
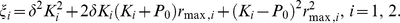
Note that the fixed point *P*_2_^*^, if stable, involves the outcompetition of the second viral strain, *x*_2_, by the first one, *x*_1_; while *P*_3_^*^ involves the reverse scenario, that is, the virus population *x*_1_ is outcompeted by the *x*_2_ population whenever this is a stable fixed point. The stability of these two equilibria was numerically studied under the parameter ranges shown in Fig. 4 (see below).

We note the existence of another fixed point involving the asymptotic coexistence of both viral populations, which is given by *P*_4_^*^ = (*x*_1_^*^ = *ζ*, *x*_2_^*^ = *ψ*), with
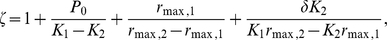
and
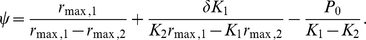



### Numerical analyses of the model using empirical estimates of accumulation rate and virulence

By studying the parameters denoting accumulation (i.e., *r_max,i_*) as well the parameters related to virulence (i.e., *K_i_*) we numerically characterized the possible scenarios of virus extinction (equilibrium *P*_1_^*^), outcompetition (equilibria *P*_2_^*^ and *P*_3_^*^) and coexistence (equilibrium *P*_4_^*^). The model qualitatively reproduced the outcome of the competition experiments discussed in the previous section. To reproduce the experimental observations TEV-PC76 we set the relative accumulation rate to *r_max_*_,1_ = 1.07 and virulence to *K*_1_ = 0.817. For TEV-PC2, we also set *r_max_*_,2_ = 0.621 and virulence to *K*_2_ = 1.221. The *K_i_* values were fixed to the empirical virulence values determined by Carrasco *et al.* [17]. The results are shown in Fig. 4(a) right, together with the representation of the equilibrium concentrations of the two viral populations using the previous values of *r_max,i_*, in the parameter space (*K*_1_, *K*_2_). The results showed that for the values of *K_i_* previously mentioned, TEV-PC76 outcompetes TEV-PC2. Actually, for these values of accumulation rate, the only combination of virulence that allows the outcompetition of TEV-PC76 by TEV-PC2 would be a lower virulence for the later, thus indicating that when two viruses are competing, a slower replicator can still outcompete a faster one if there are large differences in virulence. All the time series computed under the biologically meaningful parameter values (see Fig. 4(a), right) indicate that for all the used initial conditions of the virus populations, TEV-PC76 outcompetes TEV-PC2. This scenario involves that the fixed point *P*_2_^*^ is stable, and thus the eigenvalues obtained from det(*J*(*P*_2_^*^)−*λ* **I**) = 0, are *λ*_±_<0. For the same parameters, and extensively, to all the parametric region covered by the gridded surface of Fig. 4(a) [as well as of Fig. 4(b)], the equilibrium corresponding to the outcompetition of the second virus, *P*_2_^*^, is stable.

**Figure 5:**
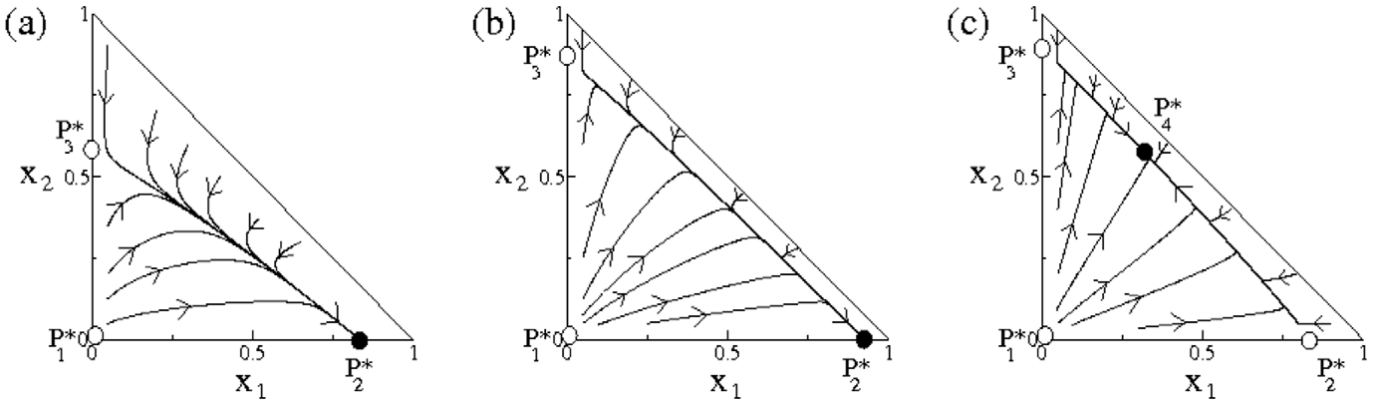
Phase portraits obtained numerically from **Eqs. (1)**–**(2)** displaying the dynamics in the phase plane (*x*_1_, *x*_2_), with *x*_1_+*x*_2_ = 1, and the stability of the fixed points: *P*_1_^*^, *P*_2_^*^, *P*_3_^*^, and *P*_4_^*^ (stable and unstable equilibria are shown, respectively, in black and white circles). In (a) we use the experimental values used in Fig. 4 (a) right. In (b) we use the same values of Fig. 4(b) left. In both cases the origin is a repeller; *P*_3_^*^ is a saddle; *P*_2_^*^ (outcompetition of *x*_2_ by *x*_1_) is stable and the equilibrium *P*_4_^*^ is outside the phase plane. In (c) we show the asymptotic coexistence scenario, where *P*_2_^*^ becomes a saddle and the fixed point *P*_4_^*^, which is stable, is inside the phase plane (here we use *r_max_*_,1_ = 1.07, *r_max_*_,2_ = 0.621, *K*_1_ = 0.8 and *K*_2_ = 0.2). The arrows in all the plots indicate the directions of the flows.

The previous results showed that a hypovirulent virus could outcompete a hypervirulent one provided that the former is a faster replicator. Actually, the effect of virulence seems to be important in the outcompetition dynamics. For instance, in the parameter space displayed in Fig. 4(a), and for some values of virulence, the slower replicating virus can outcompete the faster one, specifically for those values at which the slower replicator also has a lower virulence (see flat surface in Fig. 4(a) left). To get into this phenomenon we repeated the parameter space using *r_max_*_,1_ = 0.85 and *r_max_*_,2_ = 1. Now, as a difference from the previous analyses, the first population of viruses (*x*_1_) has a lower fitness. The results displayed in Fig. 4(b) (gridded surface) indicate that *x*_1_ outcompetes the second virus population (with a largest fitness), for low values of *K*_1_ (these values can grow when *K*_2_ also grows). In Fig. 4(b) we show several time series using different initial conditions with *K*_1_ = 0.2<*K*_2_ = 0.7, (also with *r_max_*_,1_ = 0.85 and *r_max_*_,2_ = 1) where the slower replicator outcompetes the fastest one. We also analyzed numerically the effect of the initial conditions (using different values of the initial conditions with *x*_1_(0)+*x*_2_(0) = 1) on the asymptotic dynamics found in the parametric regions studied in Fig. 4 (results not shown). These analyses revealed that almost all the initial conditions reach the equilibria found in Fig. 4 (i.e., *P*_2_^*^ in the gridded surface and *P*_3_^*^ in the flat surface), thus indicating that scenarios of bistability are very unlikely. To illustrate the dynamics and the basins of attraction we represent in Fig. 5 three phase portraits, which show the dynamics for several initial conditions in the phase plane (*x*_1_, *x*_2_). We specifically used the same parameter values used in Figs. 4(a) and 4(b), also adding another parametric scenario with coexistence between both virus populations (Fig. 5c). This coexistence scenario, although arising in a small region of parameter space (Fig. 4a), might involve the existence of polymorphisms in multiplication rate and virulence. Hence, two different evolutionary strategies could stably coexist within a single host. The fixed points previously characterized analytically as well as their stabilities are also shown in each phase portrait (Fig. 5).

In conclusion, in agreement with the experimental results, a fast replicating hypovirulent viral strain can outcompete a hypervirulent one provided it replicates slower. Our model also shows a wide region in parameter space for which slow replicating and hypovirulent strains can still outcompete fast replicating hypervirulent ones.

## Discussion

In this work we have studied two mutants of TEV differing in virulence and multiplication rate but not in infectivity rate. The first mutant genotype (TEV-PC76) has a higher multiplication rate and is hypovirulent relative to the wildtype genotype, and the second one (TEV-PC2) has a lower fitness and is hypervirulent [17]. In our experiments we have performed short-term evolution passages measuring the virus accumulation, virulence and infectivity rate. We found a lack of correlation between these three factors, which does not fit with the tradeoff hypothesis. These results suggest that the association between virulence expression and virus accumulation is not necessary simple. Even if a positive correlation between virulence and within-host multiplication has been reported occasionally for fungi [20], [21], nematodes [22] and viruses [23] there are also numerous reports showing that multiplication and virulence are uncorrelated, or even negatively correlated, for a wide range of different kinds of parasites [24]–[27]. Moreover a study with CMV has shown that evolution of virulence during passages did not affect virus multiplication [11]. Hence there is no evidence to assume that a positive relationship between within-host multiplication and virulence is a universal trend.

The results of the evolution experiments with mixed infections indicate a rapid switch in population composition where the genotype with higher multiplication rate and hypovirulent outcompetes the hypervirulent one but with lower multiplication rate. Two different phenomena can be called to explain this observation. First, to consider that virulence is not a determining factor and it is just the replication rate what drives the result of competition. The second interpretation is that virulence plays an important role (and probably in connection with replication), and a hypovirulent virus can outcompete a hypervirulent one. In order to further explore these two hypotheses, we developed and studied a two-species Lotka-Volterra mathematical model describing the competition dynamics between two viruses considering as relevant parameters the multiplication rate and virulence. By using the relative replication rate values obtained in the experiments, as well as virulence values previously estimated for these two viruses [17], our model is able to qualitatively reproduce the experimental results, where a hypervirulent slow-replicating virus is outcompeted by a hypovirulent but fastly-replicating one. Interestingly, the model also shows that under a wide parameter region, a slow-replicating hypovirulent virus can outcompete a fast-replicating hypervirulent one. In this sense, Bremermann and Pickering [28] suggested that selection would always favor the most virulent strain. These authors analyzed a model considering some of the selective forces acting upon the reproductive rates of parasites competing within groups, assuming a positive correlation between parasite's infectivity (and pathogenicity) and parasite's reproductive rate. However, our theoretical results show that slow replicating viruses may get a benefit from having low virulence. Numerical investigations of the mathematical model also indicate that the initial population numbers of both co-infecting viruses are not important in the outcompetition dynamics because the equilibrium dynamics do not have strong dependence on the initial conditions, i.e., no different basins of attraction are found or they are extremely small. Indeed, this independence from the initial conditions on the asymptotic outcompetition may also occur in the experiments: some plants in the second passage where inoculated from those of the first passage with a population almost entirely constituted by TEV-PC2 (i.e., the fraction of TEV-PC76 might be extremely low). However, in the next passages all plants were dominated by the TEV-PC76 strain.

Among the model predicted asymptotic dynamics, we have characterized a small region in parameter space where the coexistence of both viral genotypes may be possible. We note that such a region allows for the existence of polymorphic viral populations containing variants that differ in virulence and accumulation rates even within the same host; or in other words, two opposed evolutionary strategies in terms of virulence and replication can coexist. Therefore, no tradeoff between virulence and replication may be at play in our pathosystem.

Our results suggest that if hypervirulent but slow replicating and hypovirulent but fast replicating strains (or coinfecting plant viruses with differential replicative and virulence properties) had to evolve in nature, a rapid extinction of the hypervirulent would take place due to differences in accumulation rates. Indeed, if we assume an equal mutation rate for both viruses with a similar rate of infectivity, the quick switch to a pure hypovirulent population may not let any chances for a possible recombination event or time enough for TEV-PC2 to evolve by itself into a faster replicator while still retaining high virulence.

## Materials and Methods

### *In vitro* RNA transcription and inoculation

The pTEV-7DA infectious clone, kindly provided by Prof. James C. Carrington (Oregon State University), was used as our surrogated wildtype [29]. Infectious clones for the mutant strains TEV-PC2 and TEV-PC76 were generated by Carrasco *et al.* [17]. Infectious plasmids were linearized with *Bgl*II (Fermentas) and transcribed into 5′-capped RNAs using SP6 mMessage mMachine® Kit (Ambion Inc.). Transcripts were precipitated (1.5 volumes of DEPC-treated water, 1.5 volumes of 7.5 M LiCl, 50 mM EDTA), collected and resuspended in DEPC-treated water [17]. RNA integrity and quantity was assessed by gel electrophoresis and its concentration spectrophotometrically quantified with a Nanodrop. The infectivity of RNA transcripts was assessed for three viral genotypes: the wildtype TEV-7DA, the TEV-PC2 (mutation T158G of P1 cistron) and the TEV-PC76 (mutation T6519C of NIa-Pro cistron) strains. In short, sets of four weeks old *Nicotiana tabacum* cv. Xanthi plants were inoculated by abrasion of the third true leaf with 4 µg of 5′-capped RNA produced by *in vitro* transcription using mMESSAGE mMACHINE® SP6 kit (Ambion Inc.) for the first passage (Fig. 1). Plants were maintained in the green house at 25°C and 16 h light for one week. Symptoms appeared 4 to 5 days post-inoculation (dpi).

### Virus extraction

Seven dpi inoculation infected plants were collected (except the inoculated leaf) and 2 mL of extraction buffer (0.5 M borate, 0.15% thioglycollate sodium, pH 8) per gram of tissue added. Whole plant where sampled to avoid the random effects associated with bottleneck colonization of different leafs by different viral subpopulations. After homogenization, 1 mL of CHCl_3_ and CCl_4_ each were added per gram of sample, then mixed. After centrifugation (10000 g, 20 min, 4°C), the upper aqueous phase was taken and filtered through Miracloth (Calbiochem). Precipitation of viral particles was done by adding 0.11 volumes of a solution 40% PEG8000, 17.5% NaCl and incubation on ice with agitation for 30 min. After centrifugation (10000 g, 15 min, 4°C) supernatant was removed. Finally, the pellet was resuspended in 20 µL of buffer (0,05 M borate, 5 mM EDTA, pH 8) per gram of sample. The virus suspension is conserved at −80°C in 25% of sterile glycerol.

### Titration of the virus suspension

The evaluation of virus accumulation per gram of infected tissue was performed by inoculating serial dilutions of viral samples on *Chenopodium quinoa* leaves [30]. Four repetitions of each dilution (from 1∶2 to 1∶100) were inoculated on different leaves. Viral titers measured as the number of lesion-forming units (LFU) per µL of inoculum, were inferred from the regression of the observed number of local lesions 9 dpi.

### Experimental evolving populations

The following short-term evolution experiments were performed, each designed to assess the evolutionary stability of virulence and several fitness traits during single or multiple infections. For single infections, *N. tabacum* plants were inoculated either with TEV-7DA, TEV-PC2, TEV-PC76 or a 1∶1 mix of TEV-PC2 and TEV-PC76. Four independent evolution lineages were started on each case for two independent replications. Seven dpi, total plant tissue was homogenized as described above and virus extracted. Virus accumulation per gram of infected tissue was assessed and equal numbers of 20 LFU used to initiate the next evolution passage.

### Estimation of the infectivity (*ID*_50_)

An equal number of LFUs was taken for each virus and diluted in the range 10^−2^ to 10^−6^. Each dilution was used to inoculate 20 *N. tabacum* plants. Seven dpi all infected plants were counted. The trimmed Spearman-Karber method was used to evaluate the *ID*_50_ after the first, the third and fourth passage [31].

### Measuring virulence

Virulence was defined as the reduction in host's fitness associated with infection. Practically, this was done by quantifying the total number of germinating seeds from infected plants in relation to the number of germinating seeds produced by healthy plants [17]. This measure was performed for the TEV-7DA, TEV-PC2 and TEV-PC76 at the first and last evolution passages.

### Discrimination of viral genotypes by restriction analysis

Restriction enzymes Eco81I and EcoT14I were used to check which viral genotype was present at each evolution passages. Eco81I cleaves the mutant TEV-PC2 P1 sequence but not the corresponding wildtype sequence of TEV-PC76 at this locus, whereas EcoT14I cleaves the mutant TEV-PC76 NIa-Pro sequence but not the wildtype sequence found in this locus for TEV-PC2.

### Statistical analyses

The effect of genotype (main factor) and passage number (covariable), as well as their interaction, on virulence, virus accumulation and infectivity were assessed by a model II ANOVA. Both factors were treated as random ones. Prior to analyses, virus accumulation data were log-transformed to achieve normality and homoscedasticity of variances. Statistics were done with SPSS v16.
